# Detection of *Arsenophonus* and *Spiroplasma* throughout the life cycle of Colombian populations of *Spodoptera frugiperda*: evidence of potential vertical transmission

**DOI:** 10.7717/peerj.21921

**Published:** 2026-11-09

**Authors:** Anny J. Yepes-Acevedo, Natalia Lopez-Rivera, Carolina Montoya-Ruiz, Claudia Ximena Moreno-Herrera, Clara Ines Saldamando-Benjumea

**Affiliations:** 1 Departamento de Biociencias, Facultad de Ciencias, Grupo de Biotecnología Vegetal UNALMED-CIB, Laboratorio de Ecología y Evolución de Insectos, Universidad Nacional de Colombia, Medellin, Colombia; 2 Departamento de Ciencias de la Vida, Facultad de Ciencias de la Vida, Grupo de Biotecnología Vegetal UNALMED-CIB, Laboratorio de Ecología y Evolución de Insectos, Universidad Nacional de Colombia, Medellin, Colombia; 3 Departamento de Biociencias, Facultad de Ciencias, Grupo de investigación Microbiodiversidad y Bioprospección, Laboratorio de Biología Celular y Molecular, Laboratorio Procesos moleculares, Universidad Nacional de Colombia, Medellin, Colombia

**Keywords:** Fall armyworm, *Arsenophonus*, *Spiroplasma*, Insect microbiota, Vertical transmission, Life-history traits

## Abstract

**Background:**

*Spodoptera frugiperda*, commonly known as the fall armyworm (FAW), is a pest of great economic importance in corn. The species has recently expanded from the Western Hemisphere to Africa, Asia, and Oceania, increasing its global impact. FAW has diverged into two strains, corn and rice, which differ in genetics, reproductive isolation, resistance to insecticides and to *Bacillus thuringiensis*, and, more recently found, in their microbiota. Endosymbionts such as *Arsenophonus* and *Spiroplasma* have been detected using next-generation sequencing (NGS) of the 16S rRNA gene. However, information on additional endosymbionts and their transmission dynamics remains limited. Therefore, the objectives of this study were to (i) evaluate the presence of endosymbionts such as *Arsenophonus, Cardinium*, *Spiroplasma*, and *Wolbachia* as well as the intracellular parasite Microsporidia, throughout the life cycle of both strains of *S. frugiperda*, (ii) assess their potential vertical transmission, and (iii) examine possible associations between these symbionts and life-history traits in this insect.

**Methods:**

Larvae were sampled from corn and rice fields in central and eastern Colombia. Laboratory colonies of the corn and rice strains of *S. frugiperda* were maintained under controlled conditions. Strain identity was confirmed using diagnostic molecular markers (*MspI*, *SacI*, FR). Within each strain, one-to-one crosses were performed to construct life cycle tables and estimate life-history traits components. DNA was subsequently extracted from parental individuals and from eggs, larvae, pupae, and adults. Following extraction, conventional and real-time polymerase chain reaction (PCR) using an intercalating dye were conducted to identify the genera *Arsenophonus, Cardinium*, *Spiroplasma*, and *Wolbachia*, the intracellular parasite Microsporidia and positive amplifications were confirmed by Sanger sequencing.

**Results:**

*Arsenophonus* was detected exclusively in the corn strain using conventional PCR and confirmed by Sanger sequencing. Additionally, *Spiroplasma* was detected in both strains using real-time PCR using an intercalating dye. The detection of these endosymbionts across developmental stages is consistent with, vertical transmission. Differences in life-history traits components were observed between the two strains, but no clear association was detected between these differences and the presence of either endosymbiont under the conditions evaluated. These results highlight the need for further studies to evaluate the potential role of endosymbionts in the biology of this pest and to further on to better understand their transmission dynamics and possible ecological interactions.

## Introduction

*Spodoptera frugiperda (J.E. Smith)*, commonly known as the fall armyworm, is a lepidopteran native to the Western Hemisphere and considered one of the most significant agricultural pests worldwide (Overton et al., 2021; Yainna et al., 2022). Its high capacity for migration has made it allowed to expand from America (Juárez et al., 2012; Durand, Yainna & Nam, 2022; Tay et al., 2023) to Africa (Goergen et al., 2016), Asia (Nagoshi et al., 2020) and Oceania (Nayyar et al., 2021), and its polyphagous behavior allows it to feed on around 353 host plants, including crops of great economic importance such as corn, rice, sorghum, sugar cane and cotton (Saldamando & Vélez-Arango, 2010; Montezano et al., 2018; Nurkomar et al., 2023), which had generated significant losses in the Agricultural globally production (Ayil-Gutiérrez et al., 2018; Overton et al., 2021; Tambo et al., 2023). In Colombia, *S. frugiperda* outbreaks cause severe defoliation, yield losses, and increased pesticide use, challenging sustainable agriculture (Cano-Calle, Arango-Isaza & Saldamando-Benjumea, 2015).

In addition to its wide distribution and host ranges, *S. frugiperda* exhibits a complex population structure, with two distinct strains: the corn strain and the rice strain. Both have been identified by molecular markers, including polymerase chain reaction (PCR)-restriction fragment length polymorphism (RFLP) assay targeting the mitochondrial cytochrome c-oxidase subunit I (COI) gene, digestion with restriction enzymes *MspI* and *SacI*, polymerase chain reaction (PCR) of the nuclear *S.* *frugiperda* gene (named FR), and sequencing of the triose phosphate isomerase gene (*Tpi*) (Nagoshi, Meagher & Hay-Roe, 2012; Cano-Calle, Arango-Isaza & Saldamando-Benjumea, 2015). These strains are morphologically similar but genetically differentiated (Prowell, McMichael & Silvain, 2004), and they have preferences for different host plants, particularly corn and rice (Fiteni et al., 2022). Although both can coexist in some regions, hybridization processes have been documented with proportions of up to 16% (Fiteni et al., 2022), which are accompanied by pre and post reproductive isolation, fitness reduction, and lower fertility compared to pure strains (Vélez-Arango et al., 2008; Velásquez-Vélez, Saldamando-Benjumea & Ríos-Diez, 2011; Saldamando-Benjumea et al., 2014).

The control of *S. frugiperda* has been limited by increasing resistance to chemical insecticides and toxins derived from *Bacillus thuringiensis* (Bt), prompting the search for alternative management strategies, especially in Colombia (Ríos-Díez & Saldamando-Benjumea, 2011; Ríos-Díez, Siegfried & Saldamando-Benjumea, 2012; de Almeida et al., 2017; Castañeda-Molina et al., 2023). In this context, studying insect microbiota in natural populations has emerged as a promising approach to understanding host–microbe interactions and developing alternative pest-control strategies. Symbionts play important roles in insect growth, development, nutrition, immunity, metabolism, and environmental adaptation, and may influence traits associated with pest success, including survival, reproduction, host plant utilization, and susceptibility to chemical insecticides and biological control agents, such as endotoxins produced by *Bacillus thuringiensis* (Bt) (Castañeda-Molina et al., 2023).

Characterizing the microbiota of wild insect populations offers key insights into the microbial communities driving host life-history traits and ecological adaptation. These associations help explain why populations vary in their ability to tolerate environmental stressors, exploit diverse host plants, or resist control strategies. Furthermore, identifying key microbial symbionts involved in essential processes, such as digestion, detoxification, and immune regulation, can reveal novel targets for pest management. Consequently, disrupting beneficial interactions, altering microbiome composition, or exploiting symbiont-mediated functions represent promising approaches to enhance biological control and develop more sustainable management strategies (Haider et al., 2025; Basit et al., 2025).

Accordingly, interactions between insects and their endosymbionts are increasingly integrated into modern pest management practices. Understanding these relationships supports the development of highly specific, environmentally sustainable control approaches, ultimately reducing reliance on conventional insecticides (Chaves, Neto & Tenreiro, 2009; de Almeida et al., 2017).

However, the ecological and biological consequences of these associations depend on the stability, distribution, and transmission dynamics of endosymbionts within insect populations. Particularly in pest species with complex life histories and genetic differentiation, such as *S. frugiperda*, understanding whether symbionts persist across developmental stages and strains represents an essential first step to evaluate their potential contribution to host adaptation, reproduction, and population dynamics.

Especially, maternally transmitted bacterial endosymbionts have received special attention due to their ability to influence the biology and reproduction of host insects (Kaltenpoth et al., 2025). Among them, *Wolbachia* is one of the most extensively studied symbionts and is characterized by its ability to manipulate host reproduction through mechanisms such as cytoplasmic incompatibility, parthenogenesis induction, feminization, and male-killing (Qu et al., 2013). This endosymbiont has been detected in several Lepidoptera species using PCR and sequencing approaches (Duplouy & Hornett, 2018). Within the genus *Spodoptera*, *Wolbachia* infections have been reported in *Spodoptera exempta* and *Spodoptera litura*, where molecular analyses confirmed the presence of maternally inherited strains, expanding the evidence of these associations among noctuid moths (Graham et al., 2012; Salunkhe et al., 2014). Additionally, *Wolbachia* has been detected in *S. frugiperda*, where surveys based on molecular markers have revealed the presence of this endosymbiont in natural populations, highlighting the potential relevance of *Wolbachia* host interactions in an economically important pest species (Liu et al., 2024). More recently, *Spodoptera picta* was also identified as a host for a novel *Wolbachia* strain (wSpic); genomic and transcriptomic evidence suggests a stable association with possible effects on host reproductive traits, including ovarian development and fecundity (Niu et al., 2025). These findings expand the known diversity of *Wolbachia* associations within *Spodoptera* and emphasize the importance of investigating how this endosymbiont may influence host biology, adaptation, and population dynamics.

In addition to *Wolbachia*, other endosymbionts, such as *Arsenophonus, Spiroplasma, Cardinium*, and Microsporidia, have been reported in various arthropods. Microsporidia are obligate intracellular parasites that infect numerous invertebrates and some vertebrates (Gupta et al., 2016). It has been reported in *Anopheles gambiae*, where its vertical transmission has been documented (Nattoh et al., 2023), and in the silkworm *Bombyx mori*, where it can affect larval and pupal development (Gupta et al., 2016). *Cardinium* has been detected in various arthropods, including the parasitoid *Plagiomerus diaspidis* and the genus *Brevipalpus* in mites, where its involvement in parthenogenetic reproduction has been suggested (Groot & Breeuwer, 2006; Matalon et al., 2007). Also, *Arsenophonus* induces male-killing in insects such as the parasitoid wasp *Nasonia vitripennis*, affecting up to 80% of male progeny (Duron, Wilkes & Hurst, 2010; Taylor et al., 2011; Qu et al., 2013). Likewise, bacteria of the genus *Spiroplasma* can manipulate the host reproduction, inducing male-killing in species such as *Danaus chrysippus Ostrinia zaguliaevi* and *Drosophila willistoni* (Jiggins et al., 2000; Tabata et al., 2011; Anbutsu & Fukatsu, 2011).

In Colombia, *Arsenophonus* sp. has been reported in the gonads of adult males and females of the corn strain, as determined by PCR with specific primers (Castañeda-Molina et al., 2023). In addition, recent studies based on sequencing of the 16S rRNA gene throughout the life cycle of *S. frugiperda* in the corn and rice strains have revealed partial differences in the composition of the microbiota, with *Spiroplasma* more frequently associated with the rice strain and *Arsenophonus* with the corn strain across developmental stages (Marulanda-Moreno et al., 2024).

Despite growing evidence on the role of endosymbionts in insect biology, their diversity, distribution, and transmission dynamics across developmental stages and host strains remain poorly understood in *S. frugiperda*. Therefore, this study aimed to characterize the occurrence of endosymbionts across the life cycle of *S. frugiperda* in the corn and rice strains, in order to evaluate potential vertical transmission patterns and determine whether their presence varies according to developmental stage and host strain. The analysis focused on the genera *Arsenophonus*, *Cardinium*, *Spiroplasma*, *Wolbachia*, and the intracellular parasite Microsporidia, using molecular approaches including conventional PCR, real-time PCR using an intercalating dye and Sanger sequencing.

This study provides a detailed characterization of endosymbiont occurrence across developmental stages and strains of *S. frugiperda*, contributing to a better understanding of their distribution and potential transmission patterns in this pest species. These findings constitute a baseline for future studies aimed at exploring host–symbiont interactions and their potential implications for the population biology and adaptive processes of this economically important insect.

## Materials and Methods

### Ethics statement

Larval collection and access to genetic resources were authorized by the Ministerio de Ambiente y Desarrollo Sostenible and the Autoridad Nacional de Licencias Ambientales (ANLA) to the Universidad Nacional de Colombia under Resolution No. 0255 (March 14, 2014), License No. 2025E1019464 (April 22, 2025), and the collection and access to genetic resources license No. 2025E1027997 (June 6, 2025). All procedures involving *Spodoptera frugiperda* were reviewed and approved by the Institutional Committee for the Care and Use of Animals (CICUA), Universidad Nacional de Colombia, Medellín Campus, under ethical approval CICUA-28-25.

### Insect collection and breeding stock

*Spodoptera frugiperda* larvae (*N* = 100–150) of the corn strain were individually collected from maize plants in El Carmen de Viboral (6°04′55′N, 75°20′03′W), Antioquia, northeastern Colombia, during 2024–2025. Similarly, rice strain larvae were sampled from rice fields in Arauca (7°00′16.1′N 70°56′42.7′W), department of Arauca, between October and November 2025 (Fig. 1).

**Figure 1:**
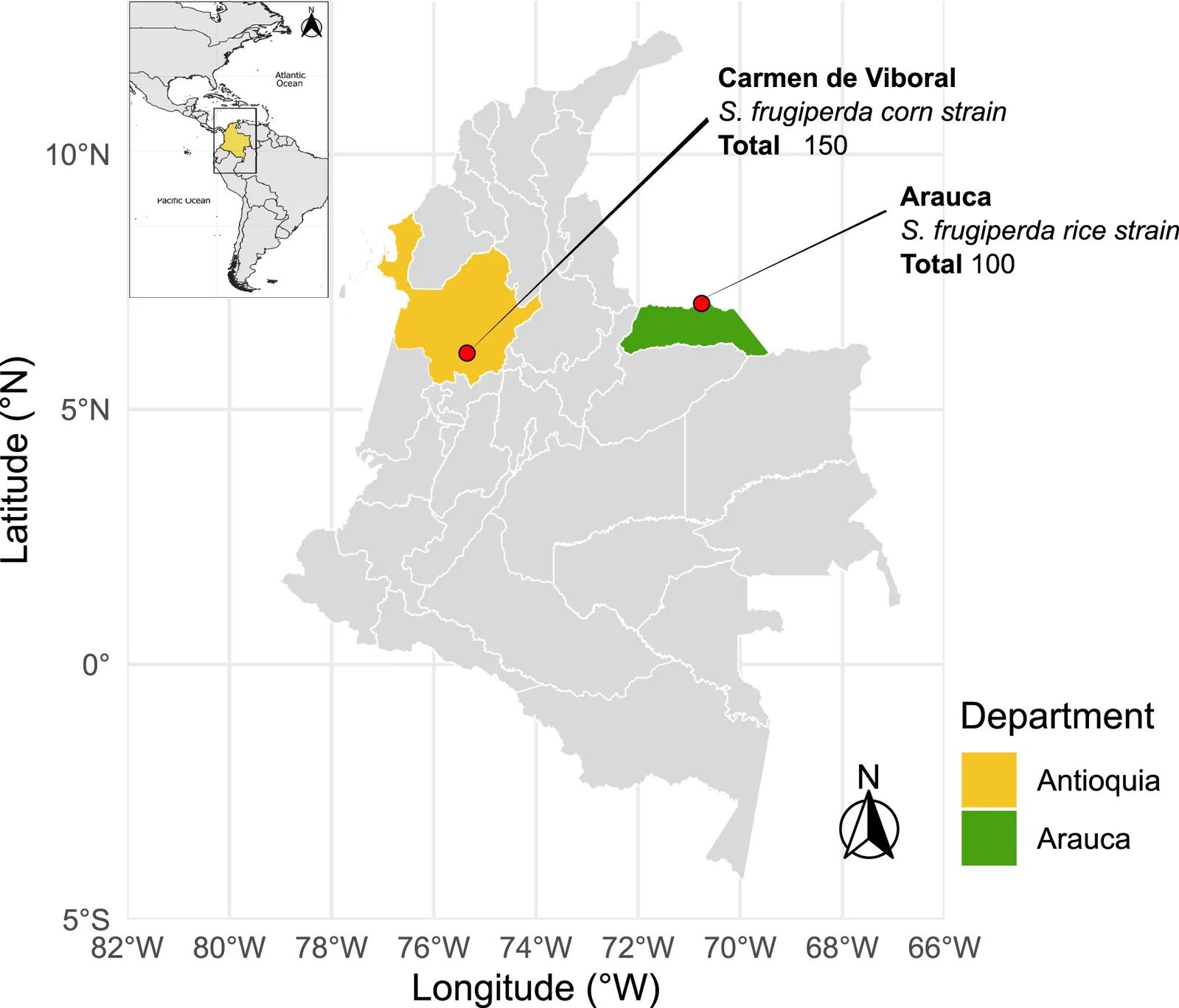
Sampling locations of *S. frugiperda* strains in Colombia. Geographic distribution of the sampling sites for *S. frugiperda* strains in Colombia. The corn strain was collected from maize plants in El Carmen de Viboral (Antioquia), while the rice strain was sampled from rice fields in Arauca (Arauca).

Collected larvae from both strains were separately reared and transported to the Laboratory of Ecology and Evolution of Insects at the Universidad Nacional de Colombia (Medellín, Colombia). Preliminary strain identification was based on morphological characters following (Stehr, 1978) and larvae were fed a bean-based artificial diet following a modified protocol (Shorey & Hale, 1965). Adults were provided with a sterile 1:1 honey solution, renewed daily, and used to obtain F_2_ generations for endosymbiont and life-history analyses. Colonies were maintained at 25 ± 2 °C, 70 ± 5% RH, with a 12:12 h light: dark cycle.

### Molecular identification of *S. frugiperda* corn and rice strains

Genomic DNA was individually extracted using a modified cetyltrimethylammonium bromide-based extraction method (CTAB) protocol based on Raeder & Broda (1985) from parental females and their F₁ progeny (eggs, larvae, pupae, and adults). Samples from each developmental stage were individually processed and macerated in liquid nitrogen; in adults specifically, the wings were removed to improve DNA yield. Each sample was homogenized in 700 μL of extraction buffer (100 mM Tris-HCl, pH 8.0; 1.4 M NaCl; 0.02 M EDTA; 2% CTAB), and 4 μL of β-mercaptoethanol was added prior to incubation in a water bath at 65 °C for 45 m. Tubes were gently inverted every 5 min during incubation.

Following homogenization, 700 μL of chloroform: isoamyl alcohol (24:1) was added, and samples were centrifuged at 12,000 rpm for 20 min at 4 °C. The aqueous phase was transferred to a 1.5 mL microcentrifuge tube, and an equal volume (700 μL) of 100% chloroform was added. Phases were separated by centrifugation at 12,000 rpm for 10 min at 4 °C. The final aqueous phase was transferred to a new tube, mixed with an equal volume (700 μL) of isopropanol, and incubated at −20 °C for 1 h prior to centrifugation at 12,000 rpm for 15 min at 4 °C.

The resulting DNA pellet was washed twice: first with 1 mL of 70% ethanol and subsequently with 1 mL of 90% ethanol, each wash followed by centrifugation at 12,000 rpm for 10 min at 4 °C. After the final wash, pellets were air-dried in an inverted position at room temperature for 20 min. DNA was resuspended in approximately 50–100 μL of 1× TE buffer. To remove residual RNA, 0.8 μL of RNase A (1 mg/mL) was added to each sample, followed by incubation at 37 °C for 1 h. The concentration and purity (A260/A280 ratio) of the extracted genomic DNA were determined using a NanoPhotometer N60 (Implen, Munich, Germany). To verify DNA quality and amplifiability, the cytochrome oxidase subunit I (COI) gene of *Spodoptera frugiperda* (Levy, Garcia-Maruniak & Maruniak, 2002) was amplified as a positive control prior to endosymbiont-specific PCR assays. DNA concentrations ranged from 200 to 300 ng/µL.

*S. frugiperda* strains were identified by PCR-RFLP analysis of the mitochondrial cytochrome c oxidase subunit I (COI) gene, using the restriction enzymes *MspI* for the corn strain and *SacI* for the rice strain. Also, we performed PCR amplification of the nuclear FR region specific to the rice strain as it produces a smear pattern superior to 500 bp, following the amplification conditions described by Cano-Calle, Arango-Isaza & Saldamando-Benjumea (2015).

### Experimental crosses and life-history traits

One-to-one (corn strain ♀ × ♂ and rice strain ♀ × ♂) crosses were conducted separately for each strain using 3-day-old virgin adults to evaluate the presence of five endosymbionts throughout the progeny life cycle (Fig. 2). Four independent crosses per strain were established, and life-history traits, including preoviposition period, oviposition period, fecundity, egg hatch rate, developmental time, and sex ratio, were recorded. Statistical analyses were performed in R, testing for normality and homogeneity of variances, followed by the application of appropriate parametric (t-test, two-way ANOVA with Tukey’s HSD *post hoc* test) or non-parametric (Mann–Whitney U test, Kruskal–Wallis test) procedures.

**Figure 2:**
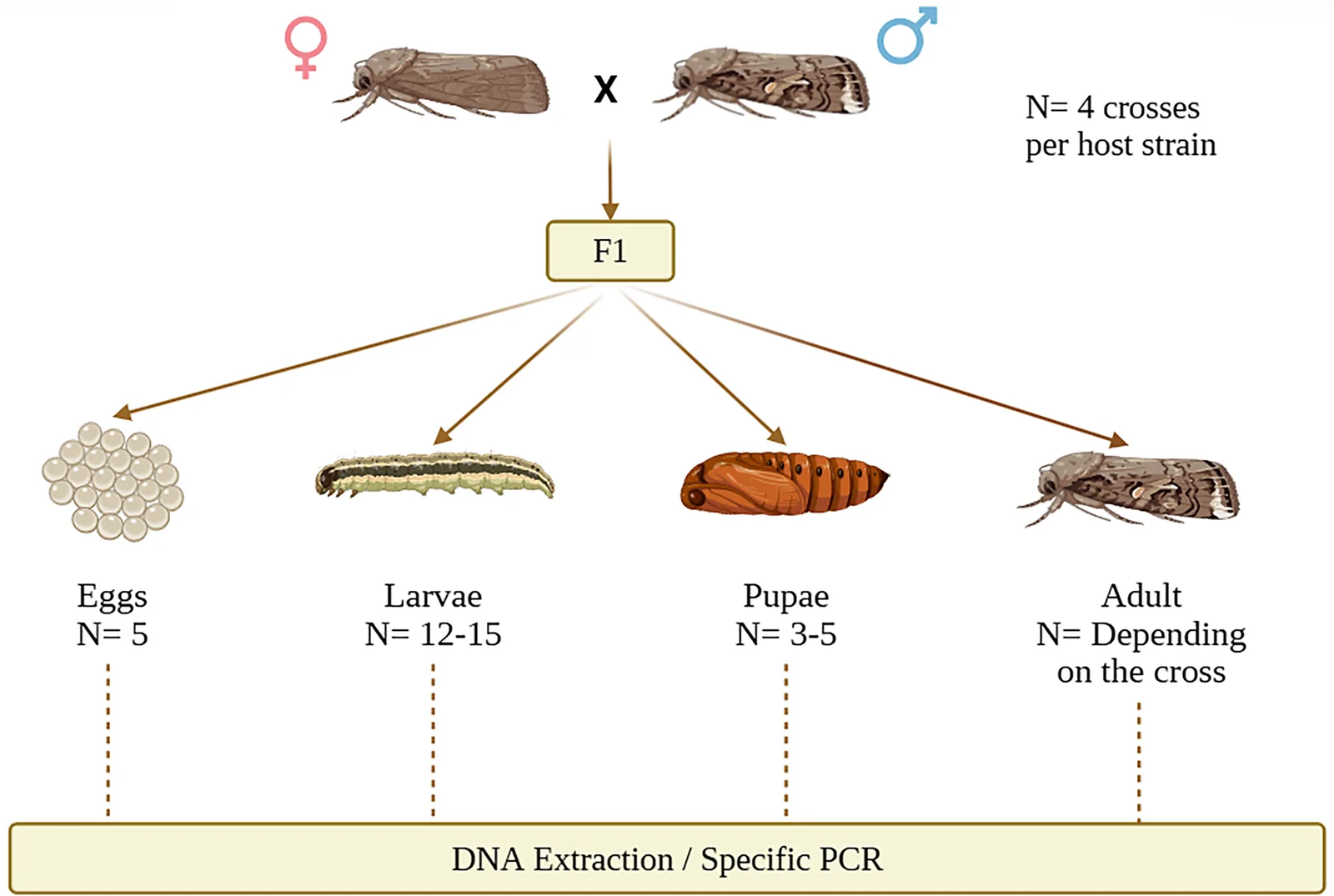
Experimental design of one-to-one crosses within corn and rice strains of *S. frugiperda*. Experimental design of one-to-one crosses (♀ × ♂) used to evaluate endosymbiont presence and life-history traits in the progeny of the corn and rice strains of *S. frugiperda*.

### Specific PCR and Sanger sequencing for the detection of endosymbionts and Microsporidia

Endosymbiont detection was performed by conventional PCR on DNA extracted from all parental individuals used in the laboratory crosses and from their F₁ offspring. For each strain and stage (eggs, larvae, pupae, adults), 10–12 individuals/instar were analyzed and were screened individually for up to five bacterial endosymbionts to assess vertical transmission in both the corn and rice strains. Remaining individuals were used for life-history traits, with maternal infections considered critical due to possible prior horizontal acquisition in the field.

DNA extracted from maternal adults of both *S. frugiperda* strains and their eggs, larvae, pupae, and adults were used to amplify five endosymbionts using previously published primers that have been standardized across diverse insect taxa. *Spiroplasma* was detected with primers Spiro1/Spiro2 (5′GGGTGAGTAACACGTATCT3′/5′CCTTCCTCTAGCTTACACTA3′; Goryacheva et al., 2018), *Arsenophonus* with ArsF/ArsR2 (5′GGGTTGTAAAGTACTTTCAGTCGT3′/5′GTAGCCCTRCTCGTAAGGGCC3′; Duron et al., 2008), *Cardinium* with CLOR1/CLOF1(5′GGAACCTTACCTGGGCTAGAATGTATT3′/5′GCCACTGTCTTCAAGCTCTACCAAC3′; Duron et al., 2008), *Wolbachia* with WSP81F/WSP691R (5′TGGTCCAATAAGTGATGAAGAAAC3′/5′AAAAATTAAACGCTACTCCA3′ ; Zhou, Rousset & O’Neill, 1998; Braig et al., 1998), and Microsporidia with SS18F/SS1492R (5′GTTGATTCTGCCTGACGT3′/5′GGTTACCTTGTTACGACTT3′; Ghosh & Weiss, 2009). PCR reactions were performed following the conditions described by Castañeda-Molina et al. (2023), with the annealing temperature set to 58 °C for *Cardinium*. Amplification products were resolved by electrophoresis on 1% agarose gels at 80 V for 45 min.

All positive PCR products were submitted for Sanger sequencing. Consensus sequences were assembled, edited, and curated using Geneious Prime 2021 v2.2 (Biomatters Ltd., Auckland, New Zealand). Endosymbiont identity was determined using BLASTn (Altschul et al., 1990). Multiple sequence alignments were generated with the ClustalW (Thompson et al., 1997) algorithm implemented in Geneious Prime 2021 v2.2. The best-fit nucleotide substitution model for the dataset was determined using the model selection function in MEGA X (Kumar et al., 2018), selecting the Kimura two-parameter (K2P) model. Bayesian phylogenetic analyses were performed in MrBayes v3.2.7 (Huelsenbeck & Ronquist, 2001). Two independent runs were conducted, each comprising four Markov chains (one cold and three heated), for 1,000,000 generations with sampling every 100 generations. The first 25% of sampled trees were discarded as burn-in. Convergence between runs was evaluated by the average standard deviation of split frequencies, which reached a final value of 0.0094, indicating adequate convergence (<0.01). Sequences of *Proteus vulgaris* (MN517893.1) and *Proteus mirabilis* (AY820623.1) were included as outgroups to root the phylogenetic tree.

### Endosymbionts detection with real-time PCR using an intercalating dye

All samples were initially screened for the presence of endosymbionts using conventional PCR. Samples yielding positive PCR results were subsequently analyzed by real-time PCR using an intercalating dye to confirm endosymbiont detection. In addition, a random subset of samples with negative conventional PCR results was also evaluated by real-time PCR using an intercalating dye to exclude potential false-negative results caused by low bacterial loads. This sequential approach was adopted because real-time PCR using an intercalating dye provides greater sensitivity than conventional PCR for detecting low-abundance endosymbionts (Weinert et al., 2015). A total of 16 DNA samples per strain (corn and rice) were analyzed, resulting in overall 32 samples, using a Gentier 48E Real-Time PCR System (Tianlong, Xi’an, China) with SYBR Green fluorescence detection and high-resolution melting curve analysis. Specific primer pairs were redesigned and synthesized to amplify conserved genomic regions of five target endosymbionts, together with a fragment of the Spodoptera frugiperda superoxide dismutase (SOD) gene, which was used as an endogenous host DNA amplification control to verify DNA quality and PCR performance (Han et al., 2021) (Table 1). A negative control (DNA-free water) and positive controls consisting of DNA containing the target regions of each bacterial endosymbiont, were included in all real-time PCR using an intercalating dye runs. The real-time PCR assay was specifically optimized for qualitative detection (presence/absence) of endosymbionts. Accordingly, assay performance was validated by demonstrating high specificity through the presence of a single melting peak, the expected amplicon size on agarose gels, and sequence confirmation of representative PCR products, providing confidence in the accuracy of target detection.

**Table 1:** Primer sequences and target genes used for real-time PCR using an intercalating dye detection of endosymbionts. Primers used for real-time PCR using an intercalating dye detection of *Wolbachia*, *Cardinium*, *Arsenophonus*, and *Spiroplasma*, and amplification of the endogenous control (*S. frugiperda SOD* gene). Target genes, primer sequences (5′–3′), and expected amplicon sizes (bp) are indicated, together with their respective references.

Organism/Control	Target gene	Sequence (5′–3′)	Amplicon size (bp)	Reference
***Wolbachia***	wsp (Wolbachia surface protein)	F-AAAATCTTTGTGAAGAGGTGATCTGC	60	Lee et al. (2012)
		R-GCACTGGGATGACAGGAAAAGG		
***Cardinium***	16S rDNA	F-CCTGGGCTAGAATGTATTTTG	133	Zhao, Zhang & Hong (2013)
		R-AAAGGGTTTCGCTCGTTATAG		
***Arsenophonus***	16S rRNA	F-TCGGTCGGGAATCCAAGG	152	Cai & Wan (2025)
		R-TCTGAGTTCCGCTCCCATC		
***Spiroplasma***	rpoB (RNA polymerase β subunit)	F-GCATACTCAACACCCGTACCA	~96	Arai, Inoue & Kageyama (2022)
		R-TGCTAACCGTGCTTTAATGGG		
***Spodoptera frugiperda*** **(endogenous control)**	SOD (mitochondrial manganese superoxide dismutase)	F-TCGGCACAATCATCAGTC	137	Han et al. (2021)
		R-AGTCCTTCTCAATAGCCTGC		

Detection of endosymbionts and the endogenous control was performed using real-time PCR with an intercalating dye, employing the Luna® Universal qPCR Master Mix (ref. M3003S, New England Biolabs). Each reaction was prepared in a final volume of 20 µL, containing 10 µL of Luna Universal qPCR Master Mix, 0.5 µL of each forward and reverse primer (10 µM), 2 µL of template DNA (approximately 200 ng), and 7 µL of nuclease-free water.

Amplification conditions consisted of an initial denaturation at 95 °C for 5 min, followed by 40 cycles of denaturation at 95 °C for 15 s, annealing at 56 °C or 60 °C for 15 s, and extension at 72 °C for 30 s. A melting curve analysis was subsequently performed from 60 °C to 95 °C to verify amplification specificity. The annealing temperature was adjusted according to the target analyzed: 60 °C for *Wolbachia*, *Arsenophonus*, and the endogenous control of *S. frugiperda*, and 56 °C for *Cardinium* and *Spiroplasma*.

Endosymbiont presence was confirmed based on Ct values exhibiting sigmoidal amplification curves before 35 cycles and melting temperatures consistent with those of the positive controls, using the endogenous *S. frugiperda* SOD gene as the reference. Representative positive real-time PCR amplicons were subsequently subjected to Sanger sequencing, and their identities were confirmed by BLASTn analysis (Altschul et al., 1990).

## Results

### Molecular identification of *S. frugiperda* corn and rice strains

*Spodoptera frugiperda* strains were identified by PCR amplification of the mitochondrial COI gene followed by restriction digestion with *MspI* and *SacI*, in combination with PCR amplification of the nuclear FR marker (Cano-Calle, Arango-Isaza & Saldamando-Benjumea, 2015). This analysis confirmed that larvae collected from maize fields in Antioquia corresponded to the corn strain (Figs. S1A, S1B), whereas larvae from Arauca were identified as rice-strain individuals and hybrids (Figs. S1C, S1D).

Molecular assays for the identification of *S. frugiperda* strains. Agarose gel electrophoresis (1%) using the GeneRuler 100 bp Plus DNA ladder. (A) PCR amplification of the nuclear FR marker in the corn strain. (B) PCR amplification of the mitochondrial COI marker followed by *MspI* and *SacI* restriction digestions in the corn strain. (C) PCR amplification of the nuclear FR marker in the rice strain. (D) PCR amplification of the mitochondrial COI marker followed by *MspI* and *SacI* restriction digestions in the rice strain.

### Experimental crosses and life-history traits

A total of eight mating pairs were established (four corn strain ♀ × ♂ and four rice strain ♀ × ♂) simultaneously per strain. Larval development time varied among crosses in both strains. In the corn strain, values ranged from 23.45 ± 3.59 to 28.12 ± 4.58 days. In the rice strain, larval development ranged from 27.10 ± 2.32 to 41.24 ± 6.90 days, with the highest value recorded in cross 4.

Pupal development time was consistent across crosses within each strain, with means near 3 days and little variability observed. In direct comparison, adult emergence time in the corn strain ranged from 8.88 ± 1.46 to 11.96 ± 2.05 days, overlapping with but slightly broader than the rice strain, which ranged from 9.95 ± 1.87 to 10.21 ± 2.42 days. Adult longevity differed more distinctly, with the corn strain generally living longer (12.10–16.88 days) than the rice strain (8.16–13.16 days) (Fig. 3).

**Figure 3:**
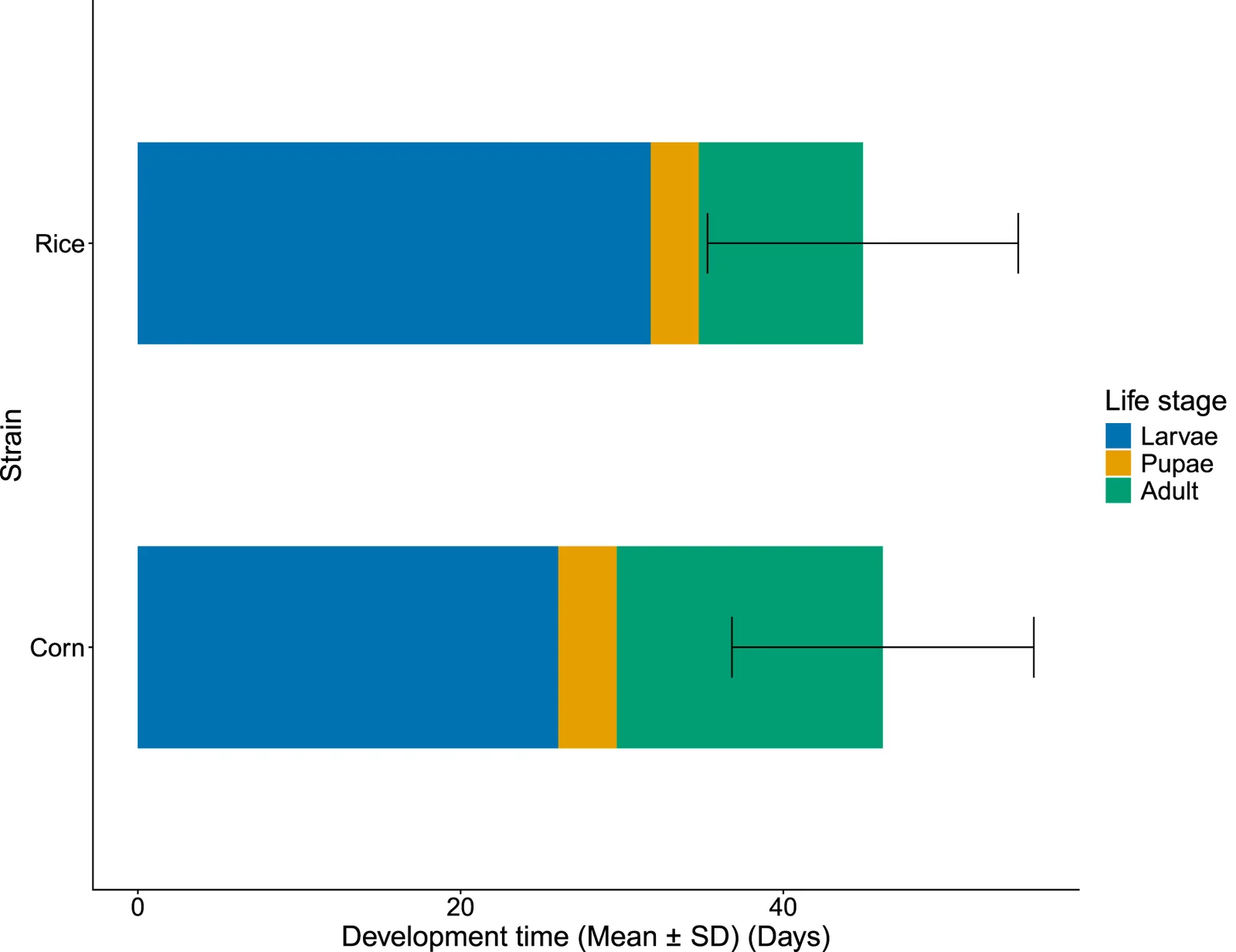
Development time of the corn and rice strains of *S. frugiperda* across different life stages. Total development time is expressed as mean ± standard deviation (days). Error bars indicate the variability in total development time for each strain.

Sex ratios were near 1:1 in most crosses; however, only cross 1 of the rice strain showed a significant female bias (1:3.75, male: female; χ^2^ = 6.36, *p* < 0.05). In contrast, other crosses did not differ significantly from the expected 1:1 ratio (*p* > 0.05) (Table S1).

Adult longevity varied considerably between strains. Most sex × strain groups did not meet normality (Shapiro–Wilk, *p* < 0.05), but homogeneity of variances was upheld (Levene’s test: F_3,12_ = 0.627, *p* = 0.611). Two-way ANOVA showed a significant strain effect on longevity (F_1,12_ = 55.76, *p* = 7.56 × 10^−6^), while effects of sex (F_1,12_ = 1.14, *p* = 0.307) and sex × strain interaction (F_1,12_ = 1.59, *p* = 0.231) were not significant. Non-parametric aligned rank transform analysis supported the strong strain effect (F_1,12_ = 45.18, *p* = 2.13 × 10^−5^). On average, corn strain individuals lived 9.63 days longer than rice strain individuals.

The preoviposition period did not differ significantly between strains (corn: 2.5 ± 0.58 days; rice: 3.0 ± 1.15 days), as shown by both parametric (t_6_ = 0.77, *p* = 0.468) and non-parametric tests (Wilcoxon W = 10, *p* = 0.64).

In contrast to the preoviposition period, the oviposition period was significantly longer in the corn strain (11.50 ± 3.11 days) compared to the rice strain (6.25 ± 2.06 days). This difference was supported by both parametric (t_6_ = −2.81, *p* = 0.031) and non-parametric analyses (W = 0, *p* = 0.029).

Additionally, total fecundity did not differ significantly between strains. Corn strain females produced 352.75 ± 69.43 eggs and rice strain females 344.50 ± 103.63 eggs (t_6_ = −0.13, *p* = 0.899; Wilcoxon *p* = 0.885). Similarly, the number of hatched eggs showed no significant strain effect (corn: 162.00 ± 84.99; rice: 170.25 ± 76.93; t_6_ = 0.14, *p* = 0.890; Wilcoxon *p* = 1.00).

However, reproductive allocation patterns differed significantly. Specifically, the corn strain produced markedly more egg masses (16.75 ± 4.65) than the rice strain (8.75 ± 3.59). This difference was statistically significant in both parametric (t_6_ = −2.72, *p* = 0.034) and non-parametric analyses (W = 0.5, *p* = 0.042). Total fecundity and the number of hatched eggs did not differ significantly between strains (Fig. 4; Table S2).

**Figure 4:**
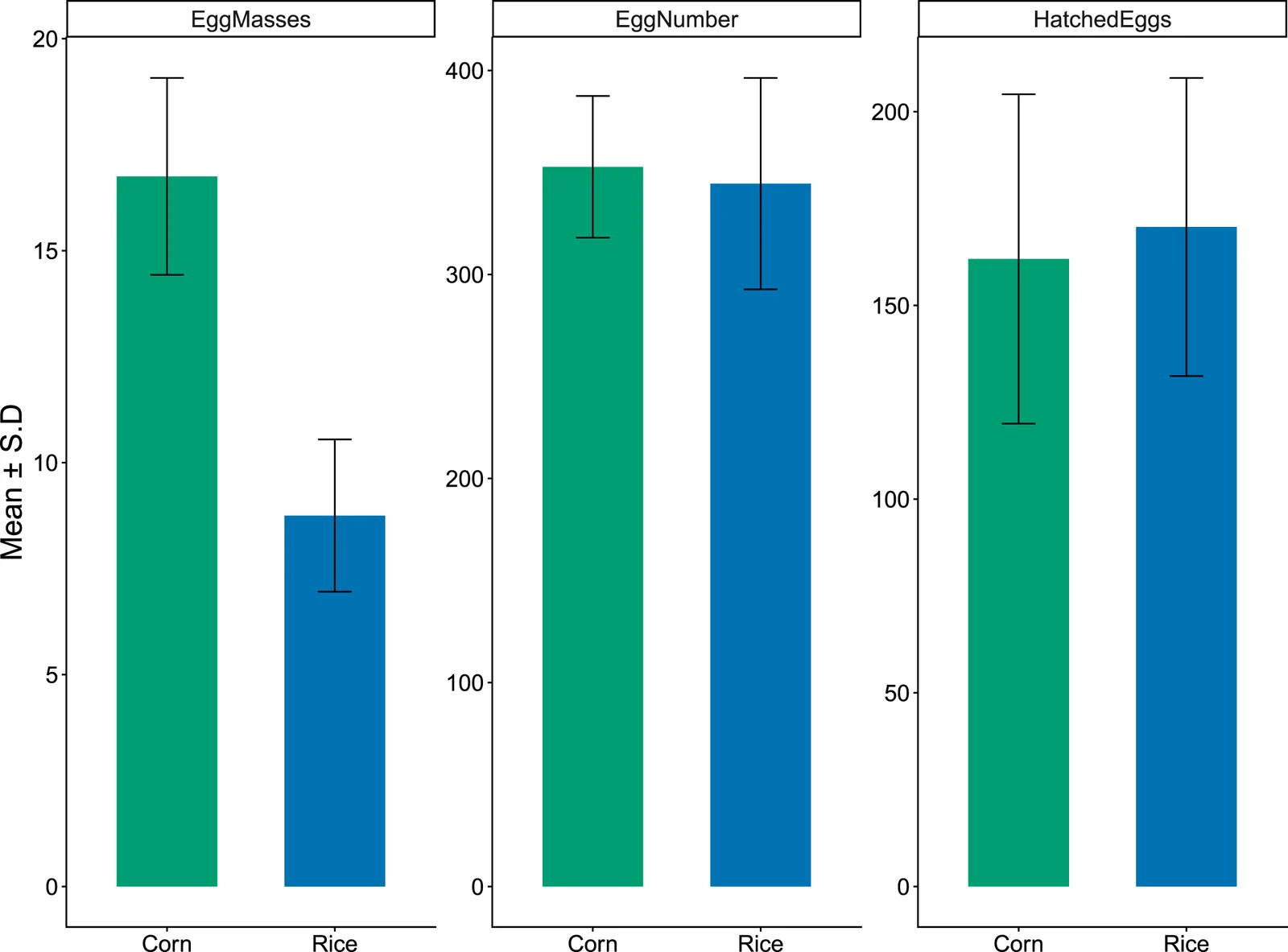
Reproductive performance of corn and rice strains of *S. frugiperda*. Reproductive output of *S. frugiperda* from the corn and rice strains. Mean (± SD) number of egg masses laid, total number of eggs, and number of hatched eggs produced by crosses of the corn and rice strains. Bars represent the mean values, and error bars indicate the standard deviation for each parameter.

Collectively, these results indicate that *S. frugiperda* strain identity primarily affects adult longevity, oviposition duration, and oviposition frequency, whereas total egg production and hatching success remain comparable across strains. The corn strain is characterized by a prolonged reproductive window and broader temporal distribution of egg laying, rather than increased per-female fecundity. No male: female distortions were observed in both strains and no association between these life-history traits components were related to an endosymbiont effect.

### Specific PCR and Sanger sequencing for the detection of four endosymbionts and Microsporidia

Genomic DNA extracted from different developmental stages showed adequate purity for downstream molecular assays. Most samples exhibited A260/280 ratios close to 2.0 and A260/230 ratios around 1.8.

DNA extracted from pupae showed the lowest quality among all developmental stages. In this stage, A260/280 ratios ranged from 0.565 to 1.372, and A260/230 ratios ranged from 0.321 to 1.882 in both host strains. These values indicate higher variability and the likely presence of residual contaminants. Nevertheless, DNA concentrations were sufficient to support perform PCR amplification for each endosymbiont and Microsporidia.

All parental females from the four experimental crosses in both host strains were screened for endosymbionts using specific PCR assays, only the female from cross 1 of the corn strain tested positive for *Arsenophonus* as evidenced by electrophoretic banding patterns (Fig. S2). In this cross, *Arsenophonus* was detected in egg masses, larvae, and adults, suggesting a consistent pattern with vertical transmission.

Agarose gel electrophoresis (1%) of PCR products obtained using *Arsenophonus*-specific primers. Mw: GeneRuler 100 bp Plus DNA ladder. Samples correspond to two adults (A46-C2; A115-C2) and one egg (H5-C3). Expected fragment size: 804 bp. **C+** represents the positive control, consisting of *Arsenophonus* DNA obtained from a different host species and included exclusively to verify the performance of the PCR assay. **C−**, negative control.

*Arsenophonus* was the endosymbiont whose presence was confirmed with Sanger sequencing, and was detected in cross 2 (eggs), cross 3 (larvae), and cross 4 (pupae). No consistent pattern of endosymbiont detection was observed among the remaining crosses. Microsporidia were not detected in any of the samples analyzed from either the corn or rice strain.

During the initial PCR screening, amplification products were obtained for *Spiroplasma*, *Cardinium*, and *Wolbachia*. However, Sanger sequencing of these amplicons produced low-quality chromatograms or sequences that did not correspond to the target endosymbionts. This result was further supported by phylogenetic analyses, in which the recovered sequences failed to cluster within the expected clades. Therefore, these amplification products were considered non-specific and were excluded from all subsequent analyses. Accordingly, only the detection of *Arsenophonus* was considered validated in these samples as sequencing outcomes were compared to Genbank data bases.

In the rice strain, PCR amplifications were unsuccessful for the screened endosymbionts, suggesting either a low bacterial load or absence of detectable infections in this population.

Taxonomic identity of the confirmed sequences was established using Basic Local Alignment Search Tool for nucleotides), available through the National Center for Biotechnology Information (NCBI) (BLASTn), which revealed sequence similarity values of 98–99%. Phylogenetic analyses showed that the obtained sequences clustered within a single, well-supported clade (Fig. 5), supporting their common classification. All *Arsenophonus* sequences with >95% identity were grouped with reference sequences of *Arsenophonus nasoniae*, including those reported by Castañeda-Molina et al. (2023).

**Figure 5:**
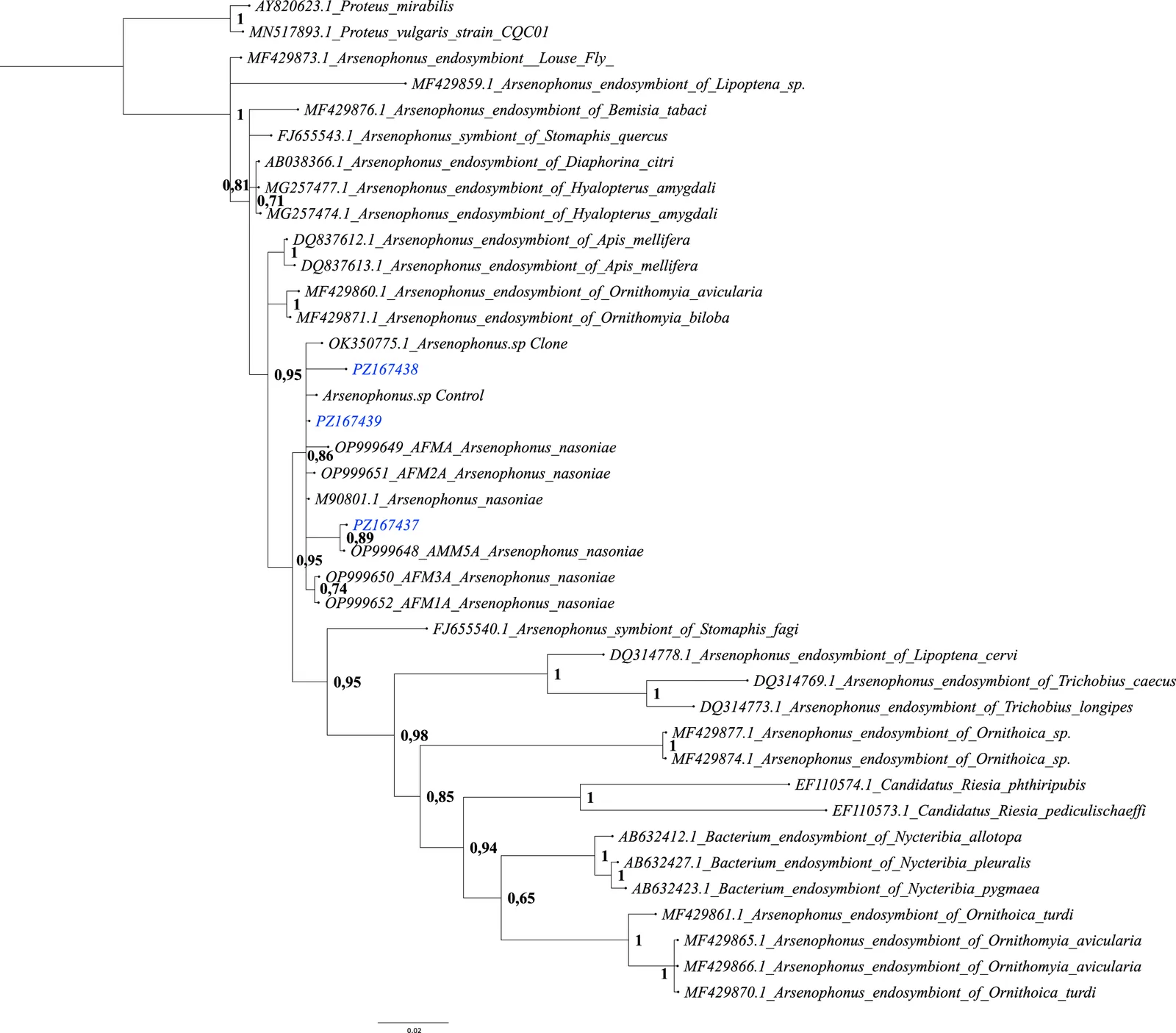
Bayesian phylogenetic reconstruction of *Arsenophonus* based on the 16S rRNA gene. The analysis was performed using the Kimura 2-parameter substitution model with 1,000,000 generations and sampling every 100 generations. Sequences of *Proteus vulgaris* (MN517893.1) and *Proteus mirabilis* (AY820623.1) were included as outgroups to root the phylogenetic tree.

None of the analyzed sequences clustered with divergent *Arsenophonus* lineages or with *Arsenophonus*-like organisms (ALO) reported from *Ornithomya* spp. (Hippoboscidae), *Trichobius* spp. (Streblidae), or primate-associated strains.

Based on their phylogenetic placement and pairwise genetic distances estimated under the Kimura two-parameter (K2P) model, the *Arsenophonus* sequences detected in *S. frugiperda* are closely related to *A.nasoniae* strains.

### Endosymbiont identification by real-time PCR using an intercalating dye

Results from the corn strain (*N* = 3 per developmental stage) included DNA extractions from the parental female and F₁ individuals, comprising egg masses, larvae, pupae, and adult males and females. Cross 1, chosen for detailed analysis, showed the presence of *Arsenophonus* in eggs, larvae, and adults, as indicated by electrophoretic banding patterns from conventional PCR. real-time PCR using an intercalating dyereturned Ct values of 26.5–30.1, indicating moderate levels of endosymbiont DNA in *S. frugiperda* samples (Fig. S3).

Initially, real-time PCR using an intercalating dye screening was performed on all parental females used in the experimental crosses. Parental females from crosses 2, 3, and 4 were also evaluated. The female from cross 2 had a coinfection of *Arsenophonus, Spiroplasma, Cardinium*, and *Wolbachia*. The female from cross 3 tested positive for *Arsenophonus* and *Spiroplasma*, whereas the female from cross 4 was positive for *Wolbachia*. These results confirm the ability of real-time PCR using an intercalating dye to detect endosymbionts in the evaluated samples.

In addition to *Arsenophonus, Spiroplasma* was also detected in the parental female and across all F₁ progeny from cross 1 (eggs:2|3, larvae:3|3, pupae: 1|3, adult: 2 |3), supporting a consistent pattern with vertical transmission in the corn strain of *S. frugiperda*. *Cardinium* was also detected in a single egg and one larval sample.

In contrast, the rice strain analysis included all parental females from four experimental crosses. Cross 2 was selected for detailed analysis because it exhibited a lower fertility rate compared to the other crosses. All parental females tested positive for *Spiroplasma*, with Ct values ranging from 31.5 to 33.8, indicating the presence of this endosymbiont in the rice strain.

*Spiroplasma* was detected in F_1_ progeny of cross 2 at different developmental stages: larvae (3|3), pupae (1|2), and adults (1|3). Ct values for these samples ranged from 29.9 to 32.2, indicating the presence of the endosymbiont in individuals descending from the positive parental female.

Sanger sequencing was obtained for real-time PCR using an intercalating dye products (~96 bp) corresponding to the *rpoB* gene of *Spiroplasma* from the rice strain. Sequences that yielded satisfactory read quality were subjected to BLAST analysis, which confirmed the identification of *Spiroplasma*. Low-quality reads were attributed to the short amplicon size and the presence of background noise in the sequencing data (Table S4).

These results indicate the persistence of *Spiroplasma* across different developmental stages in the analyzed progeny of the rice strain.

## Discussion

This study provides the first molecular evidence for the presence of endosymbionts in Colombian populations of *Spodoptera frugiperda* and confirms the persistence of *Arsenophonus* and *Spiroplasma* throughout the insects life cycle by using both conventional and intercalated PCR products. The detection of these endosymbionts across all developmental stages (eggs, larvae, pupae, and adults), as well as in the progeny of infected females, is consistent with a vertical transmission pattern. Maternal inheritance is widely recognized as a primary mechanism responsible for maintaining endosymbionts within natural insect populations, enabling their persistence across generations, fostering stable host-endosymbiont associations, and driving coevolutionary processes that shape the ecology and evolution of both organisms (Moran, McCutcheon & Nakabachi, 2008; Werren, Baldo & Clark, 2008; Hurst & Frost, 2015).

Our findings complement prior research on the microbial communities associated with *S. frugiperda*. Marulanda-Moreno et al. (2024) described the microbial diversity associated with the corn and rice strains across different developmental stages, reporting the persistence of several microorganisms throughout the insect’s life cycle. Similarly, Castañeda-Molina et al. (2023) detected *Arsenophonus*-related sequences in the reproductive tissues of *S. frugiperda*, suggesting a stable association between this bacterium and its host. The present study expands upon these observations by confirming the presence of both *Arsenophonus* and *Spiroplasma* using endosymbiont-specific molecular markers, and vertical transmission thereby providing robust evidence consistent with their intergenerational maintenance.

Interestingly, these results partially contrast with those reported by Oliveira, Rodrigues & Cônsoli (2022), who characterized the gut bacterial communities of *S. frugiperda* populations from several Latin American countries, including Colombia. Although those authors identified a similar microbiota between the corn and rice strains, they did not report *Arsenophonus*, *Spiroplasma*, *Wolbachia*, or *Cardinium* among the predominant bacterial taxa (Oliveira, Rodrigues & Cônsoli, 2022). Rather than being contradictory, these discrepancies likely reflect differences in methodological approaches and the biological components evaluated. While Oliveira, Rodrigues & Cônsoli (2022) focused on abundant gut-associated bacteria using 16S rRNA gene sequencing, our study specifically targeted endosymbiont detection in whole-body insect samples *via* conventional PCR and real-time PCR using an intercalating dye. *Arsenophonus* and *Spiroplasma* are usually found in gonads and for this reason this study was capable of detecting them. Thus, microorganisms occurring at low abundances or localized primarily within reproductive and internal tissues may escape detection in conventional gut microbiome analyses (Shao, Mason & Felton, 2024). By accounting for these methodological differences, both studies offer complementary perspectives on the microbial communities associated with *S. frugiperda* as both gut and gonads bacteria can be potential be used for biological control.

The absence of *Wolbachia*, *Cardinium*, and Microsporidia in the analyzed populations is also noteworthy. Previous studies have demonstrated that endosymbiont infection frequencies can vary considerably among insect populations, influenced by geographic origin, host genotype, environmental conditions, and host plants (Duron et al., 2008; Zhu et al., 2018). Similarly, studies and reviews focusing on Lepidoptera have shown that reproductive endosymbionts such as *Wolbachia*, *Spiroplasma*, *Cardinium*, and *Arsenophonus* exhibit heterogeneous distributions across species and populations (Ahmed, Breinholt & Kawahara, 2016; Shao, Mason & Felton, 2024). Therefore, the absence of these microorganisms in the current study does not necessarily imply their absolute absence in all Colombian populations of *S. frugiperda*, but rather indicates that their infection frequencies may vary spatially and temporally.

Among the detected microorganisms, *Arsenophonus* represents one of the most significant findings. Phylogenetic analyses clustered the retrieved sequences with lineages closely related to *Arsenophonus nasoniae*, supporting previous reports of this genus in the reproductive tissues of *S. frugiperda* (Castañeda-Molina et al., 2023). However, because the taxonomic resolution of partial 16S rRNA gene sequences depends on both the length of the amplified fragment and the variable region analyzed, taxonomic assignments were restricted to the highest taxonomic level supported by the sequence evidence (Johnson et al., 2019; Bartoš, Chmel & Swierczková, 2024). Although species-level identification would provide additional ecological and evolutionary information, it was beyond the scope of this study, which aimed to determine the occurrence and vertical transmission of the major endosymbiont genera throughout the life cycle of *S. frugiperda*. Future studies using multilocus sequence typing or whole-genome sequencing will be necessary to resolve the species and strain diversity of these endosymbionts. Members of the genus *Arsenophonus* are known to engage in a wide diverse array of interactions with insects, ranging from reproductive parasitism to mutualistic associations (Duron et al., 2008; Nováková, Hypša & Moran, 2009). Its detection in eggs and subsequent developmental stages suggests that this bacterium could be stably maintained *via* maternal transmission (Nováková, Hypša & Moran, 2009; Hurst & Frost, 2015). However, despite its persistence throughout development, the biological role of *Arsenophonus* in *S. frugiperda* remains unknown. Consequently, further studies are required to evaluate its tissue distribution, infection dynamics, and interactions with host physiology to determine whether this association carries ecological or adaptive significance.

Similarly, *Spiroplasma* was detected throughout the life cycle of both host strains. This outcome aligns with prior research characterizing *Spiroplasma* as a predominantly vertically transmitted endosymbiont capable of persisting across multiple insect generations (Haselkorn, 2010). In Lepidoptera, *Spiroplasma* has been linked to various effects, including protection against natural enemies, life-history traits modifications, and reproductive manipulation through phenomena such as male-killing (Jiggins et al., 2000; Haselkorn, 2010). Nevertheless, these effects are not universal and appear to depend on the host species, the specific bacterial strain, and environmental conditions (Shao, Mason & Felton, 2024). In this study, despite detecting *Spiroplasma* in both strains, no evidence of sex-ratio distortion was observed. This suggests that if reproductive effects do exist, they are too subtle to be detected under the evaluated experimental conditions.

Life-cycle monitoring revealed differences between the two strains regarding variables such as adult longevity, oviposition period duration, and oviposition frequency. However, these differences cannot be directly attributed to the presence of endosymbionts. *S. frugiperda* host strains exhibit well-documented genetic, behavioral, and ecological differences (Nagoshi, 2016; Marulanda-Moreno et al., 2024); hence, multiple factors could contribute to the observed variation in biological traits. Although several studies have demonstrated that endosymbionts can influence development, reproduction, nutrition, and stress tolerance in other insect-microorganism systems (Feldhaar, 2011; Zhu et al., 2018), our results do not establish a causal relationship between infection status and the biological performance of the evaluated populations. Therefore, any interpretation regarding the influence of *Arsenophonus* or *Spiroplasma* on these differences must be considered preliminary.

Furthermore, the observed sex ratios remained close to the expected 1:1 ratio in most analyzed crosses. This outcome contrasts with the patterns commonly associated with endosymbionts capable of inducing male-killing, feminization, cytoplasmic incompatibility, or other forms of reproductive manipulation (Werren, Baldo & Clark, 2008; Hurst & Frost, 2015). The absence of these phenotypes suggests that the detected endosymbionts do not exert apparent reproductive effects in the studied populations. Similar observations have been reported in other insect-endosymbiont systems, where these microorganisms persist without causing obvious phenotypic consequences under laboratory conditions (Feldhaar, 2011; Shao, Mason & Felton, 2024). Their ecological importance might only manifest under specific environmental conditions, such as pathogen exposure, nutritional stress, or interactions with parasitoids and predators (Zhu et al., 2018; Shao, Mason & Felton, 2024).

Current research on insect microbiomes emphasizes that the biological significance of associated microorganisms cannot always be inferred solely from their presence or prevalence. Some endosymbionts may persist as neutral associates or context-dependent symbionts, expressing their effects only under specific ecological conditions (Shao, Mason & Felton, 2024). In this regard, the presence of *Arsenophonus* and *Spiroplasma* throughout the life cycle of *S. frugiperda* should be interpreted as evidence of stable host-microorganism associations whose functional consequences remain to be determined.

In conclusion, our results complement previous studies on the *S. frugiperda* microbiome by demonstrating that targeted molecular approaches can successfully detect endosymbionts that may otherwise go unnoticed in conventional gut microbiome surveys (Oliveira, Rodrigues & Cônsoli, 2022; Shao, Mason & Felton, 2024). The detection of *Arsenophonus* and *Spiroplasma* across different developmental stages, combined with consistent evidence of vertical transmission, expands current knowledge regarding the microbial associations of this globally important agricultural pest. Furthermore, it provides a solid foundation for future research aimed at understanding the ecological, evolutionary, and potentially applied implications of these endosymbiotic relationships.

## Conclusions

The results of this study suggest that *Arsenophonus* and *Spiroplasma* might be vertically transmitted in *S. frugiperda*. The combined use of specific PCR, real-time PCR using an intercalating dye, and sequencing proved to be a reliable approach for detecting these endosymbionts across all developmental stages. Although species-level identification was beyond the scope of this study, future analyses based on multilocus sequence typing or whole-genome sequencing will be necessary to resolve the species and strain diversity of these endosymbionts. Further studies are also required to determine whether these bacteria affect the physiology of this insect, particularly in light of previous findings in other lepidopteran species reporting male killing and sex ratio distortion, which were not supported by the present results.

## Supplemental Information

10.7717/peerj.21921/supp-1 Supplemental Information 1 Life-history traits of corn and rice strains of *S. frugiperda* across different experimental crosses. Values represent mean ± standard deviation (SD).

10.7717/peerj.21921/supp-2 Supplemental Information 2 Statistical comparisons of life-history and reproductive traits in corn and rice strains of *S. frugiperda*. Reported values correspond to test statistics and associated *p*-values for each trait analyzed.

10.7717/peerj.21921/supp-3 Supplemental Information 3 Statistical comparisons of life-history traits in corn and rice strains of *S. frugiperda*. . Reported values correspond to test statistics and associated *p*-values for each trait analyzed.

10.7717/peerj.21921/supp-4 Supplemental Information 4 Sanger sequences and BLASTn results used to confirm *Spiroplasma* identification. Sanger sequencing reads generated from qPCR amplicons (~96 bp) targeting the *rpoB* gene of *Spiroplasma* from the rice strain of *Spodoptera frugiperda*. Sequences with satisfactory read quality were analyzed using BLASTn to confirm endosymbiont identity. Samples with unsuccessful sequencing or low-quality reads are indicated. The sequences highlighted in red correspond to those obtained by Sanger sequencing.

10.7717/peerj.21921/supp-5 Supplemental Information 5 Molecular identification of corn and rice strains of *S. frugiperda*. Molecular assays for the identification of *S. frugiperda* strains. Agarose gel electrophoresis (1%) using the GeneRuler 100 bp Plus DNA ladder. (A) PCR amplification of the nuclear FR marker in the corn strain. (B) PCR amplification of the mitochondrial COI marker followed by *MspI* and *SacI* restriction digestions in the corn strain. (C) PCR amplification of the nuclear FR marker in the rice strain. (D) PCR amplification of the mitochondrial COI marker followed by *MspI* and *SacI* restriction digestions in the rice strain.

10.7717/peerj.21921/supp-6 Supplemental Information 6 Detection of *Arsenophonus* in the corn strain of *S. frugiperda*. Agarose gel electrophoresis (1%) of PCR products obtained using *Arsenophonus*-specific primers. Mw: GeneRuler 100 bp Plus DNA ladder. Samples correspond to two adults(A46-C2 ; A115-C2) and one egg (H5-C3). Expected fragment size: 804 bp. C+ represents the positive control, consisting of *Arsenophonus* DNA obtained from a different host species and included exclusively to verify the performance of the PCR assay. C−, negative control.

10.7717/peerj.21921/supp-7 Supplemental Information 7 Melting curve analysis obtained by real-time PCR using an intercalating dye for the detection of bacterial endosymbionts in *Spodoptera frugiperda*. (A) *Arsenophonus* in the corn strain; (B) *Wolbachia* in the corn strain; (C) *Spiroplasma* in the corn strain; (D) *Cardinium* in the corn strain; and (E) *Spiroplasma* in the rice strain. Each curve corresponds to an individual positive sample. The presence of a single melting peak supports the specificity of amplification for each primer pair.

10.7717/peerj.21921/supp-8 Supplemental Information 8 The Minimum Information for Publication of Quantitative Real-Time PCR Experiments.

10.7717/peerj.21921/supp-9 Supplemental Information 9 Code for the statistical analyses performed in R. Including data processing, statistical analyses, and figure generation.

10.7717/peerj.21921/supp-10 Supplemental Information 10 Life table data for all crosses.

10.7717/peerj.21921/supp-11 Supplemental Information 11 Datasets and R scripts used for statistical analyses.

## Additional Information and Declarations

### Field Study Permissions

The following information was supplied relating to field study approvals (*i.e*., approving body and any reference numbers):

Larval collection and access to genetic resources were authorized by the Ministerio de Ambiente y Desarrollo Sostenible and the Autoridad Nacional de Licencias Ambientales (ANLA) to the Universidad Nacional de Colombia under Resolution No. 0255 (March 14, 2014), License No. 2025E1019464 (April 22, 2025), and the collection and access to genetic resources license No. 2025E1027997 (June 6, 2025).

### Data Availability

The following information was supplied regarding data availability:

The data and code are available in the Supplemental Files.

The raw sequence data are available at NCBI: PZ167437, PZ167438, PZ167439.
