# Supplementary material for: Detection of Arsenophonus and Spiroplasma throughout the life cycle of Colombian populations of Spodoptera frugiperda: evidence of potential vertical transmission
Source: PeerJ. 2026 Nov 9;14:e21921. doi: 10.7717/peerj.21921 (PMC13757732; doi:10.7717/peerj.21921)
Supplement: Supplementary file 1 [file peerj-14-21921-s005.pdf]

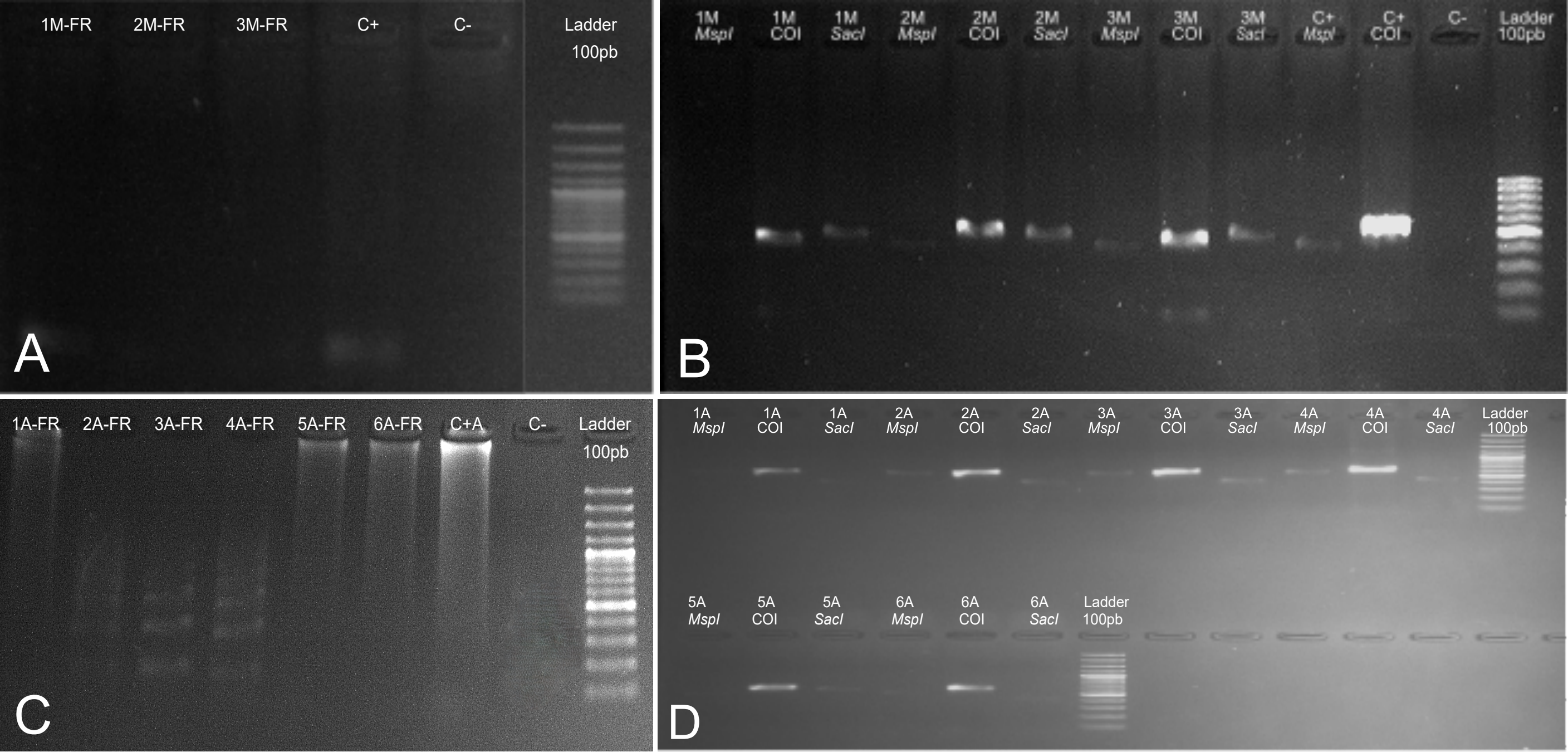
A
1M-FR
2M-FR
3M-FR
C+
C-
Ladder 100pb
B
1M MspI
1M COI
1M SacI
2M MspI
2M COI
2M SacI
3M MspI
3M COI
3M SacI
C+ MspI
C+ COI
C-
Ladder 100pb
C
1A-FR
2A-FR
3A-FR
4A-FR
5A-FR
6A-FR
C+A
C-
Ladder 100pb
D
1A MspI
1A COI
1A SacI
2A MspI
2A COI
2A SacI
3A MspI
3A COI
3A SacI
4A MspI
4A COI
4A SacI
Ladder 100pb
5A MspI
5A COI
5A SacI
6A MspI
6A COI
6A SacI
Ladder 100pb
