# Supplementary material for: Detection of Arsenophonus and Spiroplasma throughout the life cycle of Colombian populations of Spodoptera frugiperda: evidence of potential vertical transmission
Source: PeerJ. 2026 Nov 9;14:e21921. doi: 10.7717/peerj.21921 (PMC13757732; doi:10.7717/peerj.21921)
Supplement: Supplementary file 3 [file peerj-14-21921-s007.pdf]

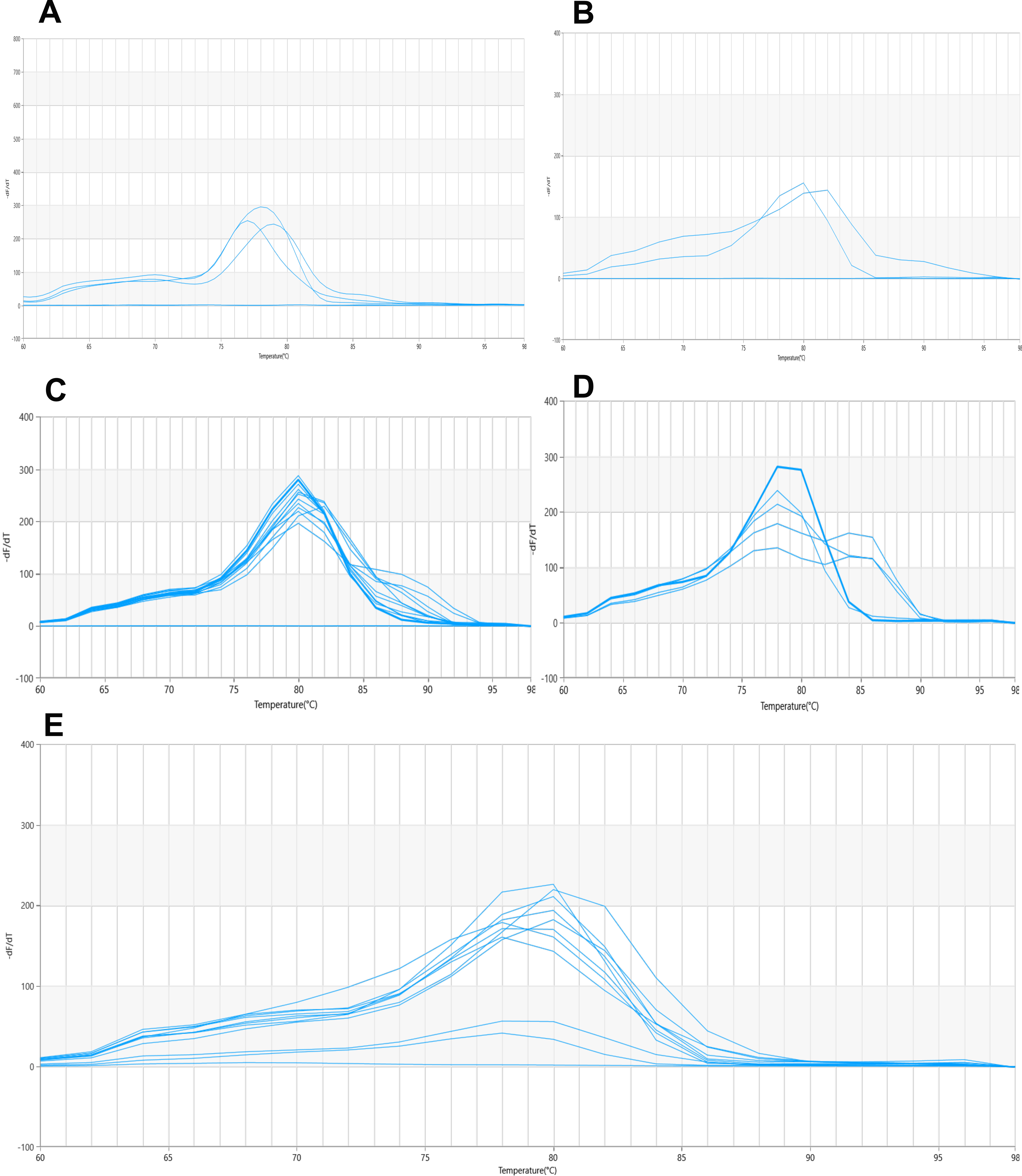
A
B
C
D
E
-dF/dT
Temperature(°C)
400
300
200
100
0
-100
60
65
70
75
80
85
90
95
98
