# Supplementary material for: Detection of Arsenophonus and Spiroplasma throughout the life cycle of Colombian populations of Spodoptera frugiperda: evidence of potential vertical transmission
Source: PeerJ. 2026 Nov 9;14:e21921. doi: 10.7717/peerj.21921 (PMC13757732; doi:10.7717/peerj.21921)
Supplement: Supplementary file 4 [file peerj-14-21921-s001.docx]

**Table S1: Life-history traits of corn and rice strains of *S. frugiperda* across different experimental crosses***.* Values represent mean ± standard deviation (SD).

|  | **Cross** | **Statistic** | **Larvae development time** | **Pupae development time** | **Adult emergency time** | **Adult longevity** | **Males** | **Females** | **Sex ratio**  ♀ × ♂ |
| --- | --- | --- | --- | --- | --- | --- | --- | --- | --- |
| Corn | 1 | Mean | 26.59 | 3.29 | 11.96 | 16.41 | 21 | 13 | 1.62:1 |
|  |  | SD | 5.88 | 0.96 | 2.05 | 11 |  |  |  |
|  | 2 | Mean | 28.12 | 2.8 | 10.2 | 12.1 | 4 | 6 | 2:3 |
|  |  | SD | 4.58 | 1.23 | 2.97 | 9.01 |  |  |  |
|  | 3 | Mean | 23.45 | 4.61 | 11.67 | 14.75 | 9 | 9 | 1:1 |
|  |  | SD | 3.59 | 2.68 | 1.78 | 8.84 |  |  |  |
|  | 4 | Mean | 26.08 | 4.07 | 8.88 | 16.88 | 5 | 8 | 1:1.6 |
|  |  | SD | 3.36 | 1.79 | 1.46 | 7.47 |  |  |  |
| Rice | 1 | Mean | 27.1 | 3 | 9.95 | 8.16 | 4 | 15 | 1:3.75 |
|  |  | SD | 2.32 | 0 | 1.87 | 5.43 |  |  |  |
|  | 2 | Mean | 28.15 | 3 | 10 | 9.33 | 6 | 9 | 2:3 |
|  |  | SD | 2.68 | 0 | 1.46 | 7.28 |  |  |  |
|  | 3 | Mean | 30.31 | 2.93 | 10.07 | 9.73 | 8 | 8 | 1:1 |
|  |  | SD | 3.32 | 0.37 | 0.8 | 8.03 |  |  |  |
|  | 4 | Mean | 41.24 | 3 | 10.21 | 13.16 | 6 | 12 | 1:2 |
|  |  | SD | 6.9 | 0 | 2.42 | 4.03 |  |  |  |
