# Supplementary material for: Detection of Arsenophonus and Spiroplasma throughout the life cycle of Colombian populations of Spodoptera frugiperda: evidence of potential vertical transmission
Source: PeerJ. 2026 Nov 9;14:e21921. doi: 10.7717/peerj.21921 (PMC13757732; doi:10.7717/peerj.21921)
Supplement: Supplementary file 5 [file peerj-14-21921-s002.docx]

**Table S2: Statistical comparisons of life-history and reproductive traits in corn and rice strains of *S. frugiperda*** Reported values correspond to test statistics and associated *p*-values for each trait analyzed.

| **Strain** | **Trait** | **Normality test (Shapiro Wilk)** | **Homogeneity Variances (t-test/F-Levene)** | **Comparison test (t-test/Anova/Kruskal Wallis)** | **Mann Whitney/Tukey test** | **Wilcoxon** |
| --- | --- | --- | --- | --- | --- | --- |
| Corn & Rice | Parental longevity | F.R: W =0.629, p=0.001 | F= 0.627, p=0.611, df= 3 | F =4.55, p=0.0001,df=1.12 | FC-FR, p = 0.004 |  |
|  |  | M.R: W =0.629, p=0.001 |  | F₁,₁₂ = 45.18, p <0.001 | MC-FR, p= 0.000 |  |
|  |  | F.C: W =0.707, p=0.014 |  |  | FC-MR, p= 0.003 | -- |
|  |  | M.C: W = 0.9984, p= 0.9951 |  |  | MC-MR, p =0.000 |  |
|  | Preoviposition | Corn, W =0.728, p =0.024 |  |  |  |  |
|  |  | Rice, W =0.728, p =0.024 | t= 0.775, p= 0.468, df=6 | -- | -- | w=10, p=0.64 |
|  |  |  |  |  |  |  |
|  | Oviposition | Corn, W =0.854, p =0.240 |  |  |  |  |
|  |  | Rice, W =0.8274, p =0.161 | t= -2.815, p= 0.03, df=6 | -- | -- | w=0, **p=0.029** |
|  |  |  |  |  |  |  |
|  | Total number of eggs | Corn, W =0.892, p =0.391 |  |  |  |  |
|  |  | Rice, W =0.950, p =0.710 | t= -0.132, p= 0.899, df=6 | -- | -- | w=7, p=0.885 |
|  |  |  |  |  |  |  |
|  | Number of hatched eggs | Corn, W =0.763, p =0.050 |  |  |  |  |
|  |  | Rice, W =0.970, p =0.842 | t= 0.144, p= 0.890, df=6 | -- | -- | w=8, p=1 |
|  |  |  |  |  |  |  |
|  | Number of egg masses | Corn, W =0.962, p =0.792 |  |  |  |  |
|  |  | Rice, W =0.928, p =0.583 | t= -2.724, p= **0.034**, df=6 | -- | -- | w=0.5, **p=0.042** |
|  |  |  |  |  |  |  |
