# Supplementary material for: Detection of Arsenophonus and Spiroplasma throughout the life cycle of Colombian populations of Spodoptera frugiperda: evidence of potential vertical transmission
Source: PeerJ. 2026 Nov 9;14:e21921. doi: 10.7717/peerj.21921 (PMC13757732; doi:10.7717/peerj.21921)
Supplement: Supplementary file 6 [file peerj-14-21921-s003.docx]

**Table S3: Statistical comparisons of life-history traits in corn and rice strains of *S. frugiperda.*** Reported values correspond to test statistics and associated *p*-values for each trait analyzed.

| **Strain** | **Trait** | **Normality test (Shapiro Wilk)** | **Homogeneity Variances (t/F-Levene)** | **Comparison test (t-test/Anova/Kruskal Wallis)** | **Mann Whitney/Tukey test** |
| --- | --- | --- | --- | --- | --- |
| Corn | Larvae development | W = 0.939, p<0.05 | F= 8.342, p<0.001, df=3,268 | F= 7.951, p<0.001, df= 3,268 | C1-C3, p = 0.001 |
|  |  |  |  | X^2^= 23.891, p<0.001, df=3 | C2-C3, p < 0.001 |
|  |  |  |  |  | C3-C4, p < 0.005 |
|  | Pupae development | W = 0.858, p<0.05 | F= 2.755, p=0.048, df= 3,78 | F= 3.656, p=0.016, df= 3,78 | C1-C3, p = 0.030 |
|  |  |  |  | X^2^= 5.848, p=0.119, df=3 | C2-C3, p =0.033 |
|  | Adult emergency | W = 0.887, p<0.005 | F= 1.036, p=0.384, df= 3,53 | F= 5.303, p=0.003, df= 3,53 | C1-C4, p=0.004 |
|  |  |  |  | X^2^= 15.033, p=0.002, df=3 | C3-C4, p = 0.027 |
|  | Adult Longevity | W = 0.942, p=0.044 | F= 0.773, p=0.517, df= 3,35 | F= 0.565, p=0.642, df= 3,35 | -- |
|  |  |  |  | X^2^= 2.330, p=0.507, df=3 |  |
| Rice | Larvae development | W = 0.884, p<0.05 | F= 8.188, p<0.001, df= 3,120 | F= 76.75, p<0.001, df= 3,120 | C4-C1, p < 0.001 |
|  |  |  |  | X^2^= 82.365, p<0.001, df=3 | C4-C2, p < 0.001 |
|  |  |  |  |  | C4-C3, p < 0.001 |
|  | Pupae development | W = 0.132, p < 0.001 | F= 1.046, p=0.375, df= 3,120 | F= 1.046, p=0.375, df= 3,120 | -- |
|  |  |  |  | X^2^= 3.133, p=0.371, df=3 |  |
|  | Adult emergency | W = 0.940, p=0.003 | F= 2.239, p=0.092, df= 3,64 | F= 0.075, p=0.973, df= 3,64 | -- |
|  |  |  |  | X^2^= 0.839, p=0.840, df=3 |  |
|  | Adult Longevity | W = 0.925, p<0.001 | F= 1.082, p=0.363, df= 3,64 | F= 2.247, p=0.091, df= 3,64 | -- |
|  |  |  |  | X^2^= 10.64, p=0.014, df=3 |  |
| Corn*Rice | Larvae development | W = 0.884, p<0.05 | F= 8.188, p<0.001, df= 3,120 | F= 76.75, p<0.001, df= 3,120 | C4-C1, p < 0.001 |
|  |  |  |  | X^2^= 82.365, p<0.001, df=3 | C4-C2, p < 0.001 |
|  |  |  |  |  | C4-C3, p < 0.001 |
|  | Pupae development | W = 0.132, p < 0.001 | F= 1.046, p=0.375, df= 3,120 | F= 1.046, p=0.375, df= 3,120 | -- |
|  |  |  |  | X^2^= 3.133, p=0.371, df=3 |  |
|  | Adult emergency | W = 0.940, p=0.003 | F= 2.239, p=0.092, df= 3,64 | F= 0.075, p=0.973, df= 3,64 | -- |
|  |  |  |  | X^2^= 0.839, p=0.840, df=3 |  |
|  | Adult Longevity | W = 0.925, p<0.001 | F= 1.082, p=0.363, df= 3,64 | F= 2.247, p=0.091, df= 3,64 | -- |
|  |  |  |  | X^2^= 10.64, p=0.014, df=3 |  |
|  |  |  |  |  |  |
