# Supplementary material for: Detection of Arsenophonus and Spiroplasma throughout the life cycle of Colombian populations of Spodoptera frugiperda: evidence of potential vertical transmission
Source: PeerJ. 2026 Nov 9;14:e21921. doi: 10.7717/peerj.21921 (PMC13757732; doi:10.7717/peerj.21921)
Supplement: Supplementary file 7 [file peerj-14-21921-s004.docx]

**Table S4. Sanger sequences and BLASTn results used to confirm *Spiroplasma* identification.** Sanger sequencing reads generated from qPCR amplicons (~96 bp) targeting the *rpoB* gene of *Spiroplasma* from the rice strain of *Spodoptera frugiperda*. Sequences with satisfactory read quality were analyzed using BLASTn to confirm endosymbiont identity. Samples with unsuccessful sequencing or low-quality reads are indicated.

| **Sample ID** | **Primer** (5’–3’) *RpoB* (RNA polymerase β subunit) | **Sequence***(5’–3’) | **Closest BLASTn matches (https://blast.ncbi.nlm.nih.gov/Blast.cgi)** | ***Spiroplasma* Sanger identification** |
| --- | --- | --- | --- | --- |
| Larvae 1 | HmSpiro_RpoB388qF | ACCGTATCTACMTGGACCTAC TACCTATMTCCATGAACTCAA TTTTCTCCGTTCCCATTAAAGC ACGGTTAGCAAG | TCCCATTAAAGCACGGTTAGCA |  |
| Larvae 1 | HmSpiro_RpoB483qR | ATACGGCTGTTGATTTAAATTT GTATGTTTGTTTGTTTGTTTGGT ATGGTACGGGTGTTGAGTATGCAT | TGGTACGGGTGTTGAGTATGC |  |
| Pupae 1 | HmSpiro_RpoB388qF | GGACCTACTACCTATATCCATGA ACTCAATTTTCTTCGTTCCCATTA AAGCACGGTTAGCA | TCCCATTAAAGCACGGTTAGCA |  |
| Pupae 1 | HmSpiro_RpoB483qR | No sequence was obtained | | |
| Adult 1 | HmSpiro_RpoB388qF | No sequence was obtained | | |
| Adult 1 | HmSpiro_RpoB483qR | TCCATGTAGATGCGGCTGTAGACAT TAATTTGTATGTTTGTTTGTTTGTT TGTGTTGGTACGGGTGTTGAGTATG CTGCAA | TGTTGGTACGGGTGTTGAGTATGCTG |  |

* The sequences highlighted in red correspond to those obtained by Sanger sequencing.
